# From Resilience to Burnout in Healthcare Workers During the COVID-19 Emergency: The Role of the Ability to Tolerate Uncertainty

**DOI:** 10.3389/fpsyg.2021.646435

**Published:** 2021-04-16

**Authors:** Michela Di Trani, Rachele Mariani, Rosa Ferri, Daniela De Berardinis, Maria G. Frigo

**Affiliations:** ^1^Department of Dynamic and Clinical Psychology, and Health Studies, Sapienza University of Rome, Rome, Italy; ^2^U.O. of Clinical Psychology, Fatebenefratelli Hospital, Rome, Italy; ^3^U.O. of Obstetric Anesthesia, Clinical Risk, Fatebenefratelli Hospital, Rome, Italy

**Keywords:** COVID-19, healthcare workers, resilience, burnout, tolerance of uncertainty

## Abstract

The COVID-19 outbreak has placed extraordinary demands upon healthcare systems worldwide. Italy's hospitals have been among the most severely overwhelmed, and as a result, Italian healthcare workers' (HCWs) well-being has been at risk. The aim of this study is to explore the relationships between dimensions of burnout and various psychological features among Italian healthcare workers (HCWs) during the COVID-19 emergency. A group of 267 HCWs from a hospital in the Lazio Region completed self-administered questionnaires online through Google Forms, including the Maslach Burnout Inventory (MBI), Resilience Scale, and Intolerance of Uncertainty Scale Short Form (IU). Cluster analysis highlighted two opposite burnout risk profiles: low burnout and high-risk burnout. The high-risk group had lower resilience and greater difficulties in tolerating the uncertainty than the low-burnout group. A set of general linear models confirmed that both IU subscales, prospective and inhibition, moderated the relationship between resilience and burnout (specifically in the depersonalization dimension). In conclusion, the results showed that individual levels of resilience and one's ability to tolerate uncertainty have been significant factors in determining the impact of the COVID-19 emergency on HCWs. The use of emotional strategies that allow individuals to stay in a critical situation without the need to control it appears to protect against burnout in these circumstances.

## Introduction

The World Health Organization (WHO) declared COVID-19 as a pandemic on March 11, 2020, when infections and deaths began to increase exponentially worldwide. The first cases were reported during December, 2019, in Wuhan, China (World Health Organization, 2020); Italy was the next country to experience a severe impact. As of December, 2020, the situation continues to deteriorate, with the World Health Organization (2020) receiving reports of 66,422,058 confirmed cases of COVID-19 worldwide, including 1,532,418 deaths. Previous studies of epidemics and quarantine suggest that such an extraordinary event will have long-term effects on mental health (Maunder et al., 2006; Kisely et al., 2020).

Now nearly a year into its impact, COVID-19 has been having a tremendous impact on the quality of life for the Italian population. The nature of individual experience of the pandemic for Italians has varied depending in part on socio-demographic factors, with women and those with previously diagnosed medical conditions bearing a particularly intense burden (Epifanio et al., 2021). The mental state of the Italian population has been severely tested, and multiple studies have found a marked increase in psychological symptoms in the non-clinical population. Furthermore, the incidence of high mortality in Italy has considerably aggravated the situation by perpetuating the traumatic dimension of grieving (Bruno et al., 2020; Forte et al., 2020; Mariani et al., 2020; Castellini et al., 2021; Velotti et al., 2021).

Correspondingly, the Italian National Health System received a severe blow with personnel infected and lost. Healthcare workers (HCWs) were the first to experience this unprecedented situation of exposure to this newly identified, contagious, and serious illness and to care for the individuals who were suffering from it. In the epicenter in the Lombardy region, they very quickly began presenting with symptoms of stress, depression, and burnout (Rapisarda et al., 2020). At the end of June, 2020, Istituto Superiore di Sanità (ISS; Italy's higher institute of health) reported that 29,476 HCWs had been infected with COVID-19, which was 12.3% of the national total of 240,578 people. From the beginning of the pandemic to November, 233 doctors died from COVID-19; this data is continuously updated day by day. Since June, almost 90% of infected people in Italy have been concentrated between hospital (70.9%) and local (18.5%) settings, while the remaining 10.6% is divided between nursing homes, residences for the elderly, and other residential or outpatient care settings. The average age of infected individuals in Italy is 58.6 years; the most affected group, with a percentage of 30%, is between 60 and 69. Beyond the personal risks that HCWs are facing, they are a potential vehicle for the spread of COVID-19 (Anelli et al., 2020; Di Monte et al., 2020; Istituto Superiore di Sanità, 2020; Galbraith et al., 2021).

During the pandemic, lockdown rules have required people to reduce social interaction in order to reduce the possibility of new infections, but HCWs have been required to continue with their daily activities. While performing intensely challenging work, they have faced concerns about family members becoming infected and have been limited in their ability to find comfort among family members who may be unable or unwilling to see them due to infection concerns (Marchetti et al., 2020). Due to the exponential increase in the demand for healthcare, they face long work shifts, often with few resources and precarious infrastructure (Kisely et al., 2020; Shigemura et al., 2020) and with the requirement of wearing personal protective equipment (PPE) that may cause physical discomfort and difficulty breathing. Moreover, many HCWs were unprepared to carry out clinical interventions for patients infected with a new virus, about which little is known and for which there are no well-established clinical protocols or treatments (Di Monte et al., 2020). A substantial percentage of healthcare staff reached the cutoff values for mental disorder concerns related to distress, depression, and anxiety. The higher the incidence of COVID-19 is, the more stressed the healthcare workers have felt (Barello et al., 2020; Chen et al., 2020; Di Tella et al., 2020; Xiao et al., 2020).

Burnout, a state of depleted psychological resources, is a strong consequence of chronic exposure to stress for HCWs (Kumar, 2016; Callahan, 2019). Risk factors for clinician burnout include stressful professional experiences, increased work load, reduced quality of work, social isolation, and younger age and career stage (Murali et al., 2018). The consequences of burnout in clinicians are important both in terms of personal well-being and patient care. Burnout has been associated with a predisposition to depression and anxiety, substance abuse, increased risk of medical errors, and poor clinical decision-making (Lapa et al., 2017). In the context of the COVID-19 pandemic, HCWs will deal with traumatic patient experiences and the unexpected loss of family, friends, and colleagues. These critical events contribute to the psychological distress clinicians will face in the COVID-19 health crisis. Several studies carried out during the initial spread of COVID-19 analyzed the risk factors of job satisfaction and mental health symptoms on health workers, showing interesting cultural differences among countries. In the USA, the findings showed that burnout levels among physicians were moderate; the critical variable was job satisfaction. No specific differences emerged for gender or marital status among physicians. However, younger physicians showed less burnout than older physicians. In China, an interesting result has been found related to individuals' proximity to the epicenter of COVID-19 spread and burnout. In fact, Zhang et al. (2020a) found a strong correlation between nearness to the epicenter and level of burnout among working adults. Their results suggest that a ripple effect or a typhoon eye effect dominates, depending on an area's distance from the epicenter. The high burnout level result strictly correlated to low distance of maximum COVID-19 diffusion. However, in Turkey, Dinibutun (2020) found a different result—burnout levels among physicians who were actively involved in the fight against COVID-19 were lower than the burnout levels of the physicians who were not actively involved. In Spain, HCWs in the areas with a higher number of cases showed a higher degree of stress globally. Workers who had been in contact directly with COVID-19 patients, like those working in respiratory medicine and those with family exposure, were predominant among the most highly stressed individuals (Portero de la Cruz et al., 2020; Romero et al., 2020).

In Italy, HCWs reported relevant work-related psychological pressure, emotional burnout, and somatic symptoms (Barello et al., 2020; Marton et al., 2020). Professionals who are directly involved in the care of patients with COVID-19 reported significant work-related psychological pressure (Rapisarda et al., 2020). Even in Italy, the impact of working in the epicenter or with COVID-19 patients presented contrasting results. In fact, Trumello et al. (2020) found no interaction effects between working (or not) with patients affected by COVID-19 and working (or not) in areas with a more severe diffusion of this pandemic. In general, levels of emotional exhaustion appeared higher than the norm, and the percentage of workers with high levels of exhaustion was significantly higher than the one found in other Italian samples before the COVID-19 outbreak (Bressi et al., 2008) or in other healthcare settings during the SARS pandemic (Maunder et al., 2006). The research by Marton et al. (2020) on Italian HCWs linked psychological symptoms and burnout to primary emotions with a cognitive component including a lack of perceived control, fear for patients and for families, feeling alone, and anger. Stress and negative emotions, together with the perceived difficulties in controlling the situation, were related to mental health.

Previous researchers in pandemic situations have identified specific variables considered likely to mediate stress responses. These were as follows: confidence in support and training, pandemic self-efficacy (ability to respond adaptively), social support, and interpersonal problems (Kang et al., 2020). Provision of assistance in developing practical competencies to face the pandemic and provision of psychological support can help to prevent psychological symptoms and increase job satisfaction (Maunder et al., 2008; Aiello et al., 2011). Ramaci et al. (2020) showed that stigma positively impacts fatigue and burnout and negatively impacts satisfaction. They also found that self-efficacy appears to relate more to the processes of discrimination and satisfaction than to those of emotional reaction (fear) and negative outcomes.

Given the severity of the COVID-19 pandemic and its potential impact on HCWs, specific psychological interventions have been and continue to be developed to provide support. Specific emergency phone lines have been planned to handle requests for psychological support in the United States (Feinstein et al., 2020), as have online Balint support groups for professionals in several countries as UK and Iran (Haude, 2020; Kiani Dehkordi et al., 2020). The Chinese government has also implemented strategies to reduce the psychological burden on HCWs. These include psychological intervention teams, use of shift duties, and online platforms with medical advice (Kang et al., 2020). All research results demonstrated the importance of regular screening of medical personnel involved in treating and diagnosing patients with COVID-19, with particular focus on stress, depression, and anxiety and provision of psychological strategies for all front-line HCWs (Folkman and Greer, 2000; Xiang et al., 2020). It is clear that the critical workers who provide care during this pandemic are highly at risk in a situation with no immediate resolution. The continuous pressure of a prolonged traumatic situation has the capacity to put the entire health system in crisis.

### Aim

The general aim of this study was to explore burnout dimensions among Italian HCWs during the COVID-19 emergency and to evaluate their relationships with some psychological features (resilience and intolerance of uncertainty). We also analyzed the relationships between burnout and socio-demographic characteristics (such as gender, age, marital status, and presence of children) and some work characteristics (such as years of experience and professional activities), which can—positively or negatively—affect one's level of work stress.

Moreover, we hypothesized that intolerance of uncertainty would serve as a moderator in the relationship between resilience and burnout, since the unpredictability of the COVID-19 experience generated a great sense of uncertainty, especially in hospital workplaces.

## Materials and Methods

### Participants

The study examined HCWs from the Fatebenefratelli Hospital in Rome between March 2020 and May 2020. Fatebenefratelli Hospital was not a COVID-19-dedicated hospital at the time of the observation, but it is an important birth center with neonatal intensive care in the capital territory serving the entire Lazio region.

The sample included 111 doctors, 88 nurses, 16 midwives, 6 psychologists, 26 laboratory technicians, and 20 administrative workers, for a total of 267 participants. In order to reduce the number of variables related to participants' departmental assignments, the HCWs were divided into two subgroups, based on their assignment to emergency services or to the chronicity management and technical services of the hospital. Table 1 reports the socio-demographic and work characteristics of the sample.

**Table 1:** Socio-demographic and work characteristics of the sample.

	**Mean *N***	**SD %**
**Age**	45.170	11.990
**Gender**		
Male	103	39
Female	164	61
**Marital status**		
Single	86	32.21
Married/cohabiting	142	53.18
Separated/divorced/widower	39	14.61
**Number of children**	1.410	0.920
**Work sector**		
Emergency group	114	43
Chronicity and services group	153	57
**Years of work experience**	18.980	11.920

### Procedure

The self-report questionnaires were made available online through Google Forms. The hospital's health management office (Bioethics Service, Fatebenefratelli Hospital), after approving the research protocol, urged employees to participate in the study. The HCWs of different services accepted voluntarily and completed the informed consent and the privacy policy disclosure before beginning the questionnaires. Data collection was anonymous. The study was carried out in accordance with the code of ethics of the World Medical Association (Declaration of Helsinki) for experiments involving humans. Ethical approval was granted by the ethics committee of the Department of Dynamic and Clinical Psychology and Health Studies of Sapienza University.

### Measures

#### Socio-Demographic and Work Characteristics

The self-administered questionnaire collected data on demographic variables (age, gender, marital status, and number of children) and on characteristics of HCWs' professional activities (hospital department in which the participant works and years of work experience).

#### Maslach Burnout Inventory

The questionnaire adopted in this study to measure burnout is the Italian validation of the Maslach Burnout Inventory (Maslach et al., 1986; Sirigatti and Stefanile, 1993; MBI), composed of 22 items with a Likert scale from 0 (never) to 6 (daily). It defines burnout in three dimensions: emotional exhaustion (EE), depersonalization (DP), and personal accomplishment (PA). EE represents the depletion of one's emotional resources (e.g., “I feel used up at the end of workday”). The dimension of DP involves viewing coworkers and clients as dehumanized objects instead of people (e.g., “I feel I treat some patients as if they were impersonal objects”). Finally, PA reflects feelings of competence, productivity, and successful achievement in one's work (e.g., “I feel I'm positively influencing other people's lives through my work”). For this dimension only, a high score indicates low burnout level. In this study, Cronbach's alpha was satisfactory for all subscales: EE (α = 0.92), DP (α = 0.80), and PA (α = 0.79).

#### Fourteen-Item Resilience Scale

The 14-item Resilience Scale (RS-14) used in this study is an assessment (Wagnild, 2009) derived from the original Resilience Scale (Wagnild and Young, 1993) that is widely used in literature. Respondents were asked to state the degree to which they agree or disagree with each item on a 7-point Likert-type scale from 1 (strongly disagree) to 7 (strongly agree). In this research, we adopted the Italian version (Callegari et al., 2016; Cronbach's alpha = 0.89).

#### Intolerance of Uncertainty Scale Short Form

The Italian validation of the Intolerance of Uncertainty Scale Short Form (IUS; Lauriola et al., 2016) is composed of 12 items measured on a Likert scale from 1 (not at all agree) to 5 (totally agree). In this questionnaire, uncertainty is conceptualized as a psychological stressor that can threaten an individual's capacity to cope effectively with situations when there is little or no information. The IUS has two scales: prospective IU and inhibitory IU. The prospective scale measures both the desire for predictability and an individual's active engagement in seeking information to increase certainty. The inhibitory scale reflects avoidance of uncertainty and paralysis in the face of uncertainty. In this study, Cronbach's alpha was 0.86 for prospective IU and 0.91 for inhibitory IU.

### Data Analysis

The statistical analyses were conducted using the Statistical Package for Social Science (SPSS) version 25 for Windows (IBM, Armonk, NY, USA). Data were reported as frequencies and percentages for discrete variables and as means and standard deviations for continuous variables.

As a first step, in order to describe burnout levels of the sample, means and SD of the different MBI dimensions were reported, and frequencies of low, medium, and clinical levels were shown based on cutoff scores of the questionnaire. We also conducted a cluster analysis, which enables the categorization of participants on the basis of their profiles of responses on a selected set of variables (dimensions on the MBI in this case). This approach allows researchers to identify groups that may not emerge via classical categorizations (i.e., low, medium, or high), but that nevertheless occur and do have a meaning for participants.

Afterwards, Pearson's correlations were performed to explore the association between burnout dimensions (emotional exhaustion, depersonalization, and personal accomplishment) and psychological features (resilience and intolerance of uncertainty). Even the groups identified by the cluster analysis were compared on psychological variables and on socio-demographic and work characteristics, through one-way ANOVAs for continuous variables (intolerance of uncertainty and resilience levels, age, number of children, and years of work experience) and chi-square analysis for categorical variables (gender, marital status, emergency vs. chronicity, and services groups).

The relationships between burnout and demographic variables, such as between burnout and characteristics of HCWs' professional activities, were also analyzed using Pearson's correlation analysis for continuous variables (age, number of children, and years of work experience) and one-way ANOVAs for categorical variables (gender, marital status, emergency vs. chronicity, and services operators; in these cases, burnout dimensions were used as dependent variables).

Finally, in order to analyze whether intolerance of uncertainty had a moderating effect on the relationship between resilience and burnout, several general linear models were tested to verify principal effects and interactions between resilience and intolerance of uncertainty—total and factor scores—(as covariates) on the different dimensions of burnout (as dependent variables).

## Results

### Burnout—Levels and Profiles

Means and SD of the dimensions evaluated are reported in Table 2. Regarding MBI levels based on cutoff criteria, for emotional exhaustion, 56% of the sample showed low levels, 24% medium levels, and 20% high levels; for MBI depersonalization, 67% showed low levels, 26% medium levels, and 7% high levels, whereas on MBI personal accomplishment, 44% showed low levels, 32% medium levels, and 24% high levels.

**Table 2:** Mean and SD for each dimension evaluated.

	**Mean**	**SD**
MBI emotional exhaustion	17.553	11.330
MBI depersonalization	4.261	4.576
MBI personal accomplishment	37.786	6.661
Resilience	79.407	10.591
IU prospective	10.865	5.304
IU inhibition	3.613	3.871
IU total	14.391	8.130

*MBI, Maslach Burnout Inventory; IU, intolerance of uncertainty*.

In order to provide a description of burnout profiles adhering to the specific research context, a hierarchical cluster analysis using Ward's method was run. We then adopted the squared Euclidean distance to determine profiles of participants according to their *z* scores on each subscale of the MBI (Hair et al., 2009; Berjot et al., 2017). The hierarchical cluster analysis suggested a two-cluster solution as shown by the dendrogram. The Bayesian index criterion (Schwarz, 1978) confirmed the two-cluster solution, as the lowest value was observed for this solution. In a second step, to validate the two-cluster solution, we ran a *k*-mean cluster analysis on the numbers of clusters emerging in the hierarchical cluster analysis (Blashfield and Aldenderfer, 1988; Ransom and Fisher, 1995).

**Figure 1:**
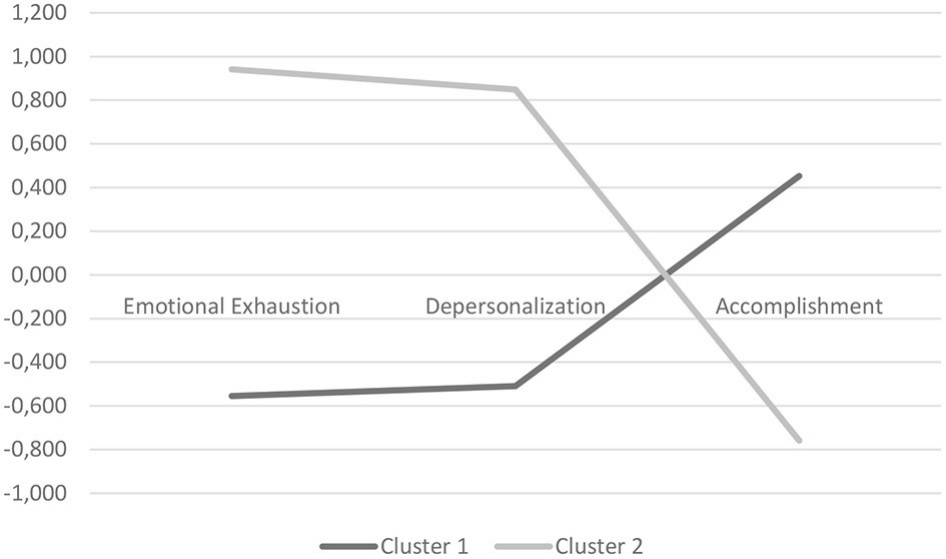
Plot of means for each variable according to clusters. Cluster 1, low burnout; cluster 2, high-risk burnout.

As shown in Figure 1, cluster 1 (labeled “low burnout” profile, *N* = 161) included healthcare personnel who had relatively low levels of emotional exhaustion and depersonalization and a higher level of personal accomplishment. Cluster 2 (“high-risk burnout” profile, *N* = 97) included healthcare personnel who had concomitantly high levels of emotional exhaustion and depersonalization and low levels of personal accomplishment.

Means and SD for each dimension of the MBI scale according to the clusters are reported in Table 3.

**Table 3:** Mean scores and standard deviations for each dimension of the MBI scale according to clusters.

	***N***	**Mean**	**SD**
**Emotional exhaustion**			
Low burnout	166	−0.555	0.567
High risk of burnout	101	0.941	0.808
**Depersonalization**			
Low burnout	166	−0.509	0.514
High risk of burnout	101	0.849	1.045
**Personal accomplishment**			
Low burnout	166	0.453	0.712
High risk of burnout	101	−0.759	0.973

### Burnout and Psychological Variables

Correlations between burnout dimensions, resilience, and intolerance of uncertainty are reported in Table 4.

**Table 4:** Pearson's correlations between burnout dimensions, resilience, and intolerance of uncertainty.

	**Resilience**	**IU prospective**	**IU inhibition**	**IU total**
MBI emotional exhaustion	−0.317^**^	0.264^**^	0.345^**^	0.330^**^
MBI depersonalization	−0.355^**^	0.262^**^	0.299^**^	0.307^**^
MBI personal accomplishment	0.473^**^	−0.102	−0.256^**^	−0.183^**^

** *p ≤ 0.01. MBI, Maslach Burnout Inventory; IU, intolerance of uncertainty. Relations between burnout, demographic variables, and characteristics of the professional activity*.

Next, we ran a series of one-way ANOVAs and chi-squares with clusters as independent variables. As shown in Table 5, significant differences emerged in both resilience and IU prospective and inhibitory. The high-risk burnout group showed significantly lower levels of resilience (*p* < 00) and higher levels of IU prospective (*p* < 00) and inhibitory (*p* < 00) than the low-risk burnout group. No differences between cluster groups emerged based on socio-demographic and work variables.

**Table 5:** One-way ANOVAs between cluster profiles on resilience and intolerance of uncertainty.

	**Low burnout**		**High-risk burnout**				
	**Mean**	**SD**	**Mean**	**SD**	***F***	***p***	**df**
Resilience	82.58	9.31	74.27	10.66	40.98	0.00	266
IU prospective	9.79	5.30	12.61	4.89	17.59	0.00	266
IU inhibitory	2.52	2.94	5.49	4.54	40.34	0.00	266

*IU, intolerance of uncertainty*.

### Burnout, Resilience, Intolerance of Uncertainty, and Socio-Demographic/Work Variables

One-way ANOVAs showed a higher level of MBI emotional exhaustion (women: *m* = 19.11, SD = 12.05; men: *m* = 15.15, SD = 9.75; *F* = 7.815, *p* = 0.006) and IU inhibition (women: *m* = 4.24, SD = 4.13; men: *m* = 2.63, SD = 3.22; *F* = 11.25, *p* = 0.001) in women than in men; whereas, men showed higher levels of resilience (women: *m* = 78.24, SD = 11.22; men: *m* = 81.09, SD = 9.27; *F* = 4.315, *p* = 0.039). No differences were found in psychological features based on marital status. Regarding the characteristics of HCWs' professional activities, we compared emergency operators vs. chronicity and services operators: a higher level of MBI personal accomplishment was found in emergency professionals than the other group (emergency group: *m* = 38.94, SD = 5.95; chronicity/service group: *m* = 35.81, SD = 8.34; *F* = 4.18, *p* = 0.006).

Correlation analysis also showed a significant negative correlation between the MBI depersonalization and age (*r* = −0.22; *p* = 0.000) and years of work experience (*r* = −0.19; *p* = 0.003) and a significant (but weak) positive correlation between MBI personal accomplishment and age (*r* = 0.16; *p* = 0.01). No significant data emerged related to number of children.

### Moderator Effect of Intolerance of Uncertainty in the Relationship Between Resilience and Burnout

Regarding the question of whether intolerance of uncertainty may moderate the relationship between resilience and burnout, results (see Table 6) showed a significant interactive effect of intolerance of uncertainty (total score) and resilience on MBI depersonalization (*B* = −0.23; *t* = −3.56; *p* = 0.00).

**Table 6:** General linear models: principal and interactive effects of resilience and intolerance of uncertainty (total score) on burnout dimensions.

	***B***	***t***	***P***
**MBI emotional exhaustion**			
Resilience	−0.274	−4.511	0.000
IU total	0.281	4.728	0.000
Resilience × IU total	−0.078	−1.141	0.255
**MBI depersonalization**			
Resilience	−0.334	−5.736	0.000
IU total	0.246	4.312	0.000
Resilience × IU total	−0.232	−3.56	0.000
**MBI personal accomplishment**			
Resilience	0.435	7.613	0.000
IU total	−0.101	−1.803	0.073
Resilience × IU total	−0.082	−1.273	0.204

*IU, intolerance of uncertainty*.

Specifically, another set of analyses—including resilience and IU factors as covariates and MBI depersonalization as dependent variable—showed a significant interactive effect of both IU prospective × resilience (*B* = −0.26; *t* = −4.02; *p* = 0.00) and IU inhibition × resilience (*B* = −0.16; *t* = −2.29; *p* = 0.02) on MBI depersonalization (see Table 7). No significant results emerged using MBI emotional exhaustion and personal accomplishment as dependent variables.

**Table 7:** General linear models: principal and interactive effects of resilience and intolerance of uncertainty (factor scores) on MBI depersonalization.

	***B***	***t***	***P***
**MBI depersonalization**			
Resilience	−0.360	−6.356	0.000
IU prospective	0.270	4.766	0.000
Resilience × IU prospective	−0.261	−4.018	0.000
**MBI depersonalization**			
Resilience	−0.324	−5.308	0.000
IU inhibition	0.162	2.671	0.008
Resilience × IU inhibition	−0.159	−2.291	0.023

*IU, intolerance of uncertainty*.

## Discussion

The COVID-19 outbreak has placed extraordinary demands upon healthcare systems worldwide. Italy is among the most severely impacted nations in terms of hospital patient overload, and its healthcare workforce struggles to cope with challenges that can threaten their well-being. The physical and psychological well-being of our HCWs are being tested as patient loads continue to increase and their fellow co-workers become infected with COVID-19, contributing significantly to burnout among healthcare workers (Patti et al., 2018; Barello et al., 2020; Di Monte et al., 2020). HCWs are also enduring significant social stigma, as they are viewed as potential transmitters of COVID-19 and therefore isolated from others (Ramaci et al., 2020). This increase in workload in the dangerous atmosphere of this pandemic has caused declining mental health among HCWs (Ayanian, 2020; Blekas et al., 2020; Lai et al., 2020; Luo et al., 2020; Marton et al., 2020; Nochaiwong et al., 2020; Pappa et al., 2020; Romero et al., 2020; Trumello et al., 2020). Thus, it is imperative that we understand the health-related consequences of the COVID-19 outbreak on HCWs to employ productive strategies to care for their mental health (Feinstein et al., 2020; The Lancet, 2020).

The general aim of this study is to explore the relationship between burnout dimensions and some psychological features, such as resilience and intolerance of uncertainty, among Italian healthcare workers during COVID-19 emergency.

Regarding burnout levels, in contrast to Barello et al. (2020) that reported a large percentage of Italian healthcare professionals with high scores in at least one of the MBI domains, in our study, only 20% of the sample had high levels of emotional exhaustion and 7% had high levels of depersonalization, whereas 44% of the sample showed high levels of personal accomplishment. HCWs still seemed to be capable of finding some gratification from their jobs, which may be considered as a relevant protective factor for the professionals' mental health, as demonstrated in previous studies (Zwack and Schweitzer, 2013; Bonetti et al., 2019). These results can be seen as part of controversial results of the impact of being in an epicenter. The hospital examined was neither a frontline treatment center for COVID-19 nor was it in the epicenter region, but it was still open for all other pathologies for a significant catchment area and for neonatal emergency care for nursing. Our results seemed consistent with previous findings that burnout is less common farther from the epicenter. However, numerous organizational changes had impacted the hospital, including the displacement of staff to other centers and the reduction of access due to the interruption of outpatient activities. The HCWs were therefore not particularly busy with the management of the pandemic, and this paradoxically may have had a frustrating effect, so that those who were most involved in the emergency (but not in an area with higher rates of contagion) reported higher personal accomplishment. In fact, the “emergency group” expressed more feelings of competence, productivity, and successful achievement in one's work than the “service operators.” These results can be compared to Dinibutun (2020) that detected lower gratification in HCWs far from the frontline. This result is consistent with the Karasek's Demand–Control theory model. According to this model, HCWs with higher level of job strain and greater decision-making responsibilities were found to be significantly more empowered, more committed to the organization, and more satisfied with their work, with lower levels of illness (Theorell and Karasek, 1996). In other words, HCWs not directly involved in the active and containing strategy of the virus presented greater stress and less control of their activities, reducing their chances of receiving gratification and dealing with the stress that the situation of uncertainty created.

Moreover, consistent with other literature (e.g., Blekas et al., 2020; Zhang et al., 2020b), burnout was more prominent in women than in men, whereas resilience was higher in men than in women. Age and years of work experience were negatively correlated with MBI depersonalization: probably, differently from other conditions, in the pandemic situation, experience played a protective role against the risk of dehumanization. These results are coherent with results from Spain where seniority was shown to be a protective factor (Romero et al., 2020).

In order to overcome the classical categorizations of participants based on cutoff scores identified in generic conditions, a cluster analysis was conducted on the MBI dimensions to identify groups with characteristics specifically related to the context examined (Berjot et al., 2017). This allowed for the identification of specific at-risk groups, which may enable the selection and deployment of specific prevention and intervention programs (Clatworthy et al., 2005). Two groups emerged, with opposite characteristics, namely, “low burnout” (low depersonalization and emotional exhaustion and high personal accomplishment) and “high-risk burnout” (high depersonalization and emotional exhaustion and low personal accomplishment). In contrast with the study of Di Monte et al. (2020) examining general practitioners, in which a third intermedial group emerged (with moderate burnout), our sample seemed to be split in two extreme groups. These two groups did not differ on socio-demographic and work-related variables, but the high-risk burnout group showed lower resilience levels and higher difficulties in tolerating uncertainty than the low-burnout group. Specifically, workers with a profile at risk of burnout presented both a tendency to desire predictability and an active engagement in seeking information to increase certainty, as well as an attitude to avoid uncertainty and to be paralyzed in the face of it. These data confirmed the previous correlation analysis, and they are consistent with findings from Di Monte et al. (2020), which reported a negative correlation between burnout and the ability to tolerate uncertainty. Results from a study by Shacham et al. (2020) of dentists and dental hygienists seem relevant to our findings as well. It may be that the between subjective overload and psychological distress could be clarified by Karasek's work demand–control–support model that claims individuals with low levels of control (along with social anxiety) are characterized by a state of confusion. It's likely that dental workers are struggling with a higher-than-normal degree of isolation, which prevents teamwork, where the unpredictable situation and unfamiliar scenarios had a strong impact on emotional distress and raised psychological defenses.

The splitting results of the cluster analysis could also indicate an effect in this population of the COVID-19 impact. The result of the cluster analysis, seen in relation to the data on resilience and uncertainty management, shows how the population reacted in facing the pandemic, increasing the fork between the risk group and the burnout-resilient group. In other words, those who were probably already in a condition of work fatigue and less personal gratification experienced the impact of the pandemic by increasing their symptomatic responses. Meanwhile, those who were more resilient and more gratified took this as an opportunity to fight the virus and cope with the situation more effectively. In this sense, the hypothesized middle groups emerged in other studies have been polarized in two more extreme reactions.

Moreover, in this condition, an individual's ability to stay in the critical situation without needing to control it and without feeling anguish in the face of uncertainty can serve as a protective factor for health. These specific characteristics can be used as indications for differentiated interventions in support of HCWs, focusing on specific individual features and pandemic reaction patterns. Strengthening individual skills is even more relevant in conditions in which the organizational level is not controllable, since, as in the case of the current pandemic, it is also in a phase of crisis and reorganization. The protective role of the ability to tolerate uncertainty is also highlighted by the interactive effect that this variable has shown with resilience in predicting burnout, in particular depersonalization. Highlighting individual resource factors, and supporting these resources through focused psychological interventions, prevents not only workers' distress but also the consequences of professional stress on work quality and on their relationships with patients.

There are several limitations inherent in the present study. First, the use of self-report questionnaires through online platform may have affected the collected data. At the time of data collection, it was not possible to recruit participants in person and have the measures administered by a clinician.

Also, since the COVID-19 pandemic affected regions of Italy in different ways, it would be interesting to have a larger sample from a wider geographic area to be able to verify whether the relationships between burnout and psychological characteristics are different depending on the severity of the health emergency in a given area. This is especially true because the study was conducted out of epicentral area.

A third limitation involves the absence of a control group, which would be useful in future investigations for performing comparative analysis with staff of COVID-19 hospitals, rather than general practitioners. The analysis was conducted in a general hospital facility that was not specifically focused on COVID-19 interventions.

In addition, long-term follow-up to collect further data on HCWs' health status would help to verify the predictive role of burnout on the long-term psycho-physical health of participants.

In conclusion, HCWs who are dealing with the current emergency in healthcare settings are the pillars of the COVID-19 epidemic response. It is therefore essential to invest as much as possible to protect their physical and mental health. Implementing psychological support resources to help those who are tackling the emergency on a daily basis and ensuring their continued availability when the emergency is over can improve coping skills and promote personal empowerment. Focusing interventions, both training and psychological support, on enhancing resilience and the ability to act in conditions of uncertainty without needing to establish control could help to provide concrete suggestions to direct actions for our HCWs.

## Data Availability Statement

The raw data supporting the conclusions of this article will be made available by the authors, without undue reservation.

## Ethics Statement

The studies involving human participants were reviewed and approved by Ethics Committee of the Department of Dynamic and Clinical Psychology, and Health Studies, Sapienza University of Rome. The patients/participants provided their written informed consent to participate in this study.

## Author Contributions

MDT contributed to all the phases of the study from conception and design of the study, results interpretation, and writing manuscript. RM performed the statistical analysis, contributed to results interpretation, and in writing the manuscript. RF contributed to conception and design of the study results interpretation. DD contributed to conception of the study, data collection and implementation of dataset, writing manuscript. MGF contributed data collection and supervision. All authors contributed to manuscript revision, read, and approved the submitted version.

## Conflict of Interest

The authors declare that the research was conducted in the absence of any commercial or financial relationships that could be construed as a potential conflict of interest.
